# Communication patterns in the doctor–patient relationship: evaluating determinants associated with low paternalism in Mexico

**DOI:** 10.1186/s12910-020-00566-3

**Published:** 2020-12-10

**Authors:** Eduardo Lazcano-Ponce, Angelica Angeles-Llerenas, Rocío Rodríguez-Valentín, Luis Salvador-Carulla, Rosalinda Domínguez-Esponda, Claudia Iveth Astudillo-García, Eduardo Madrigal-de León, Gregorio Katz

**Affiliations:** 1 Population Health Research Centre, National Institute of Public Health, Cuernavaca, Morelos, Mexico; 2 Centre for Mental Health Research, Australian National University, Canberra, Australia; 3 Research Ethics Committee, National Institute of Public Health, Cuernavaca, Morelos, Mexico; 4 Psychiatric Care Services, Ministry of Health, Mexico City, Mexico; 5 Hospital Director at the National Institute of Psychiatry Ramón de La Fuente Muñiz, Mexico City, Mexico; 6 Department of Mental Health, Faculty of Medicine, National Autonomous University of Mexico, Mexico City, Mexico

**Keywords:** Paternalism, Autonomy, Physician–patient communication, Self-determination, Shared decision-making, Patient-centred medicine

## Abstract

**Background:**

Paternalism/overprotection limits communication between healthcare professionals and patients and does not promote shared therapeutic decision-making. In the global north, communication patterns have been regulated to promote autonomy, whereas in the global south, they reflect the physician’s personal choices. The goal of this study was to contribute to knowledge on the communication patterns used in clinical practice in Mexico and to identify the determinants that favour a doctor–patient relationship characterized by low paternalism/autonomy.

**Methods:**

A self-report study on communication patterns in a sample of 761 mental healthcare professionals in Central and Western Mexico was conducted. Multiple ordinal logistic regression models were used to analyse paternalism and associated factors.

**Results:**

A high prevalence (68.7% [95% CI 60.0–70.5]) of paternalism was observed among mental health professionals in Mexico. The main determinants of low paternalism/autonomy were medical specialty (OR 1.67 [95% CI 1.16–2.40]) and gender, with female physicians being more likely to explicitly share diagnoses and therapeutic strategies with patients and their families (OR 1.57 [95% CI 1.11–2.22]). A pattern of highly explicit communication was strongly associated with low paternalism/autonomy (OR 12.13 [95% CI 7.71–19.05]). Finally, a modifying effect of age strata on the association between communication pattern or specialty and low paternalism/autonomy was observed.

**Conclusions:**

Among mental health professionals in Mexico, high paternalism prevailed. Gender, specialty, and a pattern of open communication were closely associated with low paternalism/autonomy. Strengthening health professionals’ competencies and promoting explicit communication could contribute to the transition towards more autonomist communication in clinical practice in Mexico. The ethical implications will need to be resolved in the near future.

## Background

In the doctor–patient relationship, the principle of respect for autonomy [1] implies that patients receive information from their doctors about their diagnosis, treatment and prognosis in an adequate and appropriate way that allows them to make informed decisions and that, in dialogue with their doctors, patients can choose their own degree of participation in this process [2]. In this regard, there is evidence of clinical cases in which, under certain cultural, social or religious circumstances, communicating all the available information could be harmful to patients [3]. Some patients prefer not to receive detailed information about their clinical condition, while others feel comfortable being informed of their diagnosis and some aspects of their prognosis. [4]. The conflict between patients’ right to receive all available information from their doctors and cultural factors (traditions, beliefs, values, norms, symbols and meanings shared by members of a community to varying degrees [5]) that could oppose such disclosure poses a challenge for professionals when communicating with their patients [6]. Although the need to tell the truth is unquestionable, scenarios in which patients are unwilling to allow their doctors to give them more information about their health status could lead doctors to adopt a paternalistic attitude (this implies that the doctor makes decisions based on what he or she considers best for the patient, even for those patients who could make such decisions for themselves [7]).

Three ethical perspectives have been described regarding physicians’ interaction with their patients: paternalistic, autonomist, and reciprocal. According to Beauchamp [8], paternalism is used in the ethical literature to refer “to practices that restrict individuals’ freedom, without their consent, justified by the intent to prevent any harm they would do to themselves or to produce some benefit for them that they would not otherwise obtain” [9]. Paternalism proposes that the doctor directs the care of his patient, who plays a passive role; in contrast, in the autonomist perspective, communication that leads to an informed patient is promoted [10]. The paternalistic attitude assumes that doctors always know what is best for their patients [11], while from the perspective of reciprocity, the medical staff collaborate with their patients, patients’ relatives, and others to give them a significant opportunity to participate in healthcare [12].

While respect for a patient’s autonomy is commonly observed in doctor–patient interactions in the United States, in many cultures and particularly in Latin America, the proportion of doctors and families who believe in paternalism as a form of beneficence is still significant [6]. In wanting to providing protection, physicians could voluntarily withhold information about the diagnosis and prognosis, which may interfere with and even disregard the patient’s preferences [13]. Luna [14] considers that among illiterate populations in Latin America, certain characteristics (a low educational background, leading to difficulties in making informed decisions) make communication impossible and that, physicians should therefore decide what kind of treatment the patient should receive; the same attitude has been observed toward patients from socially marginalized groups [15]. There are trends that establish the benefits of a high degree of paternalism for the US Latino populations that do not participate in clinical decisions, including the assumption that healthcare professionals can provide medical services of high ethical quality and establish a great degree of intimacy with the family [16]. There are also opinions that acknowledge that in the case of some clinical conditions, such as neuropsychiatric disorders, patient autonomy may be compromised [17]. In such situations, families’ and patients’ rights in terms of their autonomy should be one of the main aspects put forth by mental health professionals, even as a way to increase the capacity of the patient’s decision-making, which may be overly weak at times [18].

Physicians’ sociodemographic, personal and professional characteristics have an influence on doctor–patient interactions. According to some studies, paternalism increases with physician’s age [19, 20]. Gender differences have also been observed in physicians’ attitudes and communication styles, with female physicians more often engaging in dialog [21], adopting a partnership-building style [22] and tending to be autonomist [19] than male colleagues. Likewise, other factors, such as religion and physician specialty may be a source of differences in doctor–patient communication. Morita et al. found that physicians’ attitudes towards patient autonomy were significantly correlated with the physician’s specialty and with physicians having no religion but following a specific philosophy [23].

At the end of the day, the main goals for communication between physicians and their patients are to establish a good interpersonal relationship to facilitate information exchange and involve patients in decision-making [24]. In this context, we show the results of a survey on perceptions of communication patterns used by a selection of mental healthcare professionals in Central and Western Mexico. We used a questionnaire that included professional and personal reflections, case studies and clinical vignettes and questions regarding educational level and specialty. The goal of this study was to contribute to the knowledge on communication patterns used in clinical practice in Mexico and to identify the determinants that favour a low paternalistic/autonomist doctor–patient relationship.

## Materials and methods

### Participants

We conducted a survey of a convenience selection of 761 mental health professionals assisting children with intellectual development disorder (IDD), autism spectrum disorder (ASD), and attention deficit hyperactivity disorder (ADHD) to explore their personal and professional characteristics in relation to their communication patterns with the children’s parents. We invited psychiatrists, psychologists, nurses, social workers, and residents in psychiatry and related healthcare areas to participate. Healthcare professionals worked in mental, neurological and children’s hospitals in Mexico City (Fray Bernardino Psychiatric Hospital, National Institute of Neurology and Neurosurgery, Ramón de la Fuente Muñiz National Institute of Psychiatry, Dr. Juan N. Navarro Children's Psychiatric Hospital). In addition, we included residents from the Medical School of the National Autonomous University of Mexico, paediatricians from the Children’s Hospital (central State of Morelos), and psychiatrists from the psychiatric healthcare services of Mexico City and the Jalisco Institute of Mental Health. At each institution, an in-person interviewer invited mental healthcare professionals to participate. The interviewer explained the objective of the study and the fact that participation was voluntary, and that the information provided would be confidential. Subsequently, informed consent was obtained from those who agreed to participate, and they were given the questionnaire, which was collected in the following two hours or the next day. The study was carried out from June 2018 to January 2019.

### Questionnaire

"Patrones de comunicación de profesionales de la salud con padres de sujetos con: Trastorno del desarrollo intelectual (TDI), Trastorno del espectro autista (TEA), y Trastorno del déficit de atención-hiperactividad (TDAH)" ("Health professionals’ communication patterns with parents of subjects with: Intellectual development disorder (IDD), Autism spectrum disorder (ASD), and Attention deficit hyperactivity disorder (ADHD)"), an instrument in Spanish, was used [25]. The instrument contains 64 items and is composed of two sections: (a) professional and personal reflections and (b) case studies or clinical vignettes. The personal reflections section corresponds to questions that explore situations encountered by professionals in both medical care and in their daily lives; responses were used to determine attributes such as paternalism, the value that professionals place on truth, their attitudes towards death, and their communication patterns. The clinical vignettes section presents case studies and includes questions regarding diagnosis, prognosis and treatment, which were used to construct indicators of the mental health professionals’ knowledge about IDD, ASD, and ADHD. In addition, the questionnaire included variables related to educational level and specialty, as well as religion and bioethics training. The questions were answered using a Likert-type response, which allowed an understanding of mental healthcare professionals’ level of agreement with the proposed statements (strongly disagree, disagree, agree, and strongly agree). The questionnaire is an adapted version of an original instrument previously used in other studies in Mexican populations [26, 27] and has shown adequate internal consistency (0.76) through the Kuder-Richardson test [28]. When the questionnaire was developed, an expert panel assessed the relevance and clarity of the selected items after three rounds of review.

### Primary outcome

Paternalism was defined as an attitude and behaviour in which mental health professionals impose their outlooks and decisions on their patients, limiting patient autonomy with the belief that they do so for the benefit of their patients or themselves. Paternalism (dependent variable) was constructed based on the following questions:

- (a) The reaction that I want to inspire in my patients diagnosed with a chronic disease is *1*—Confidence and calmness, *2*—A combative spirit, *3*—Active participation, *4*—I do not intervene in the moods of my patients.
- (b) The best hope we can give to a parent with a child diagnosed with IDD/ASD is to make him/her feel that life can continue as normally as possible.
- (c) Emotional distress does very little; therefore, I try to assist the children’s parents as much as possible by avoiding feelings such as sadness, grief or anguish.
- (d) Enthusiasm should be shared to encourage parents, even if it means telling a lie.
- (e) We create others’ reality. For example, if a parent with a child diagnosed with an incurable disease sees that I stay calm, the parent will think, "If the physician is calm, the situation might not be so bad".
- (f) When I see someone looking crestfallen, my first reaction is to try to distract that person to encourage him/her, even if it requires changing the subject.
- (g) Talking about painful topics only makes the pain worse.
- (h) When I have a problem, I try to conceal it from my loved ones.
- (i) I was always taught to avoid causing someone distress.

First, the answers to each of the questions were addressed; those indicating that professionals were in favour of low paternalism/autonomy were considered correct. Subsequently, a score was assigned; for example, if the professional fully agreed with one of the questions that indicated low paternalism, 4 points were assigned, in accordance with the Likert scale score (strongly agree, 4 points; agree, 3 points; disagree, 2 points; strongly disagree, 1 point.) We determined the arithmetic sum of the scores for each item, and based on their distribution (tertiles), determined the following categories of paternalism: high paternalism/overprotection (T1, reference category), moderate paternalism (T2) and low paternalism/autonomy (T3).

### Independent variables

The possible paternalism predictors analysed were: (a) communication pattern, which was defined as the behaviour reported by a mental health professional in relation to his/her communication style with parents when discussing the diagnosis, prognosis and/or treatment of patients with IDD, ASD and ADHD. To construct this indicator, 11 questions from the instrument were selected based on input from experts (items 1, 2, 6, 8, 9, 10, 11, 18, 33, 34, 40); (b) Value assigned to truth, which refers to the value the healthcare professional assigned to conveying the truth in his/her communication with parents; in other words, the correspondence between what the healthcare professional knows about the situation and what the healthcare professional tells the parents (items 18, 19, 28, 31); (c) Attitude towards death, which refers to healthcare professional’s willingness to adapt, react and act in situations related to death (items 28, 29, 30, 41, 43, 44, 47, 49); (d) Family member with IDD or ASD, which asks whether any member of the professional's family has been diagnosed with IDD or ASD (item 13); (e) Bioethics courses, which refers to courses related to medical ethics that the healthcare professional has taken throughout his/her professional training (item 16); and (f) Religion, which refers to whether the professional describes him/herself as a believer or nonbeliever in terms of religion (item 53). To construct the communication pattern, value assigned to truth and attitude towards death indicators, the same methodology as for the paternalism indicator was applied.

This study also included indicators related to knowledge about IDD, ASD, and ADHD as paternalism predictors. To construct these indicators, three clinical vignettes were given in the last section of the questionnaire. Clinical vignettes were presented as cases or scenarios featuring people of a specific age with IDD, ASD or ADHD and were accompanied by different questions on diagnosis, prognosis and treatment [25]. Answers were considered correct if they were among those selected by the group of paediatric psychiatry experts. For IDD and ADHD, 3 or 4 correct answers indicated a positive attitude and a high degree of knowledge, 2 correct answers indicated intermediate knowledge, and 1 or no correct answers indicated a low level of knowledge. For ASD, 3 correct answers indicated a positive attitude and a high level of knowledge, 2 correct answers indicated an intermediate level of knowledge, and 1 or 0 correct answers indicated a low level of knowledge. The inclusion of knowledge variables is important because they relate to the formulation of an accurate diagnosis of the mental illness being studied. Having an accurate diagnosis increases health professionals’ confidence in communicating and discussing the disorder with either the patient or his or her parents [29–32].

### Statistical analysis

A descriptive analysis of the study population was carried out. Chi-square tests were used for comparisons. To evaluate the association between communication attributes and low paternalism/autonomy, a logistic ordinal multivariate model was constructed. Odds ratios (ORs) and 95% confidence intervals (CIs) were obtained. The following variables were considered possible predictors of low paternalism/autonomy: (a) age (tertiles: 43–76 years as the reference category, 30–42 years, 19–29 years); (b) gender (male, female); (c) specialty (no, yes), (d) value assigned to truth (low, moderate, high); (e) communication pattern (withholding, partial communication, open communication—understood as the communication style for which the professional obtained the highest scores, with regard to providing the most information to parents when discussing the diagnosis, prognosis and/or treatment of patients with IDD, ASD and ADHD); (f) religion (nonbeliever, believer); (g) attitude towards death (low acceptance, moderate acceptance, high acceptance); (h) family member with IDD or ASD (yes, no); (i) bioethics courses (none, ≥ 1), and (j) knowledge about IDD, ASD and ADHD (low knowledge, intermediate knowledge, and positive attitude and high knowledge). To assess the joint effects of age and communication patterns or specialty on the likelihood of presenting low paternalism/autonomy, we created the following interaction terms: (a) age (tertiles) and communication patterns (withholding, partial communication and open communication); (b) age (tertiles) and specialty (yes, no). The reference category for each interaction was “withholding and young age” and “specialty and young age”, respectively. Ordinal regression models were also adjusted by gender, having a family member with IDD or ASD, religion, value assigned to truth, participant institution (medical facility/university), knowledge about IDD, ASD, and ADHD; and bioethics courses. Differences were considered statistically significant when *p* values were < 0.05. Stata 14 software was used for all statistical analyses.

## Results

Paternalism prevailed among mental healthcare professionals in Mexico. A total of 68.7% (95% CI 60.1–70.5) of the evaluated population presented a considerable degree of paternalism (moderate and high). Furthermore, 66.5% (95% CI 63.0–69.8) of mental health professionals said that they withheld some information from their patients. Consistent with this finding, the reported value assigned to truth was low, at 50.3% (95% CI 46.7–53.9), as seen in Table 1. Similarly, among mental healthcare professionals in Central and Western Mexico, there was low knowledge about IDD (41.7% [95% CI 38.1–45.3]) and ADHD (38.9% [95% CI 35.4–42.5]). Table 1 shows the frequency of communication attributes by paternalism strata.

**Table 1:** Sociodemographic characteristics and communication attributes of health professionals stratified by paternalism, Mexico, 2018

Variables	Overall *n* = 761	High paternalism /overprotection *n* = 364 (47.8%)		Moderate paternalism *n* = 159 (20.9%)		Low paternalism/autonomy *n* = 223 (29.3%)		*p* value^b^
	*n* (%)^a^	*n*	%^a^	*n*	%^a^	*n*	%^a^	
Age								
43–76 years	229 (30.1)	117	32.1	38	23.9	68	30.5	
30–42 years	234 (30.8)	103	28.3	53	33.3	75	33.6	
19–29 years	280 (36.8)	134	36.8	65	40.9	77	34.5	0.27
Gender								
Male	310 (40.7)	168	46.2	64	40.3	74	33.2	
Female	440 (57.8)	192	52.8	91	57.2	147	65.9	0.007
Specialty								
No	328 (43.1)	191	52.5	56	35.2	76	34.1	
Yes	425 (55.9)	172	47.3	97	61.0	146	65.5	< 0.001
Family member with IDD or ASD								
Yes	132 (17.4)	65	17.9	27	17.0	40	17.9	
No	630 (81.1)	296	81.3	129	81.1	179	80.3	0.97
Communication pattern								
Withholding	315 (41.4)	233	64.0	49	30.8	31	13.9	
Partial communication	191 (25.1)	74	20.3	59	37.1	58	26.0	
Open communication	230 (30.2)	52	14.3	48	30.2	129	57.9	< 0.001
Value assigned to truth (Truthful communication from the professional to the patient)								
Low	383 (50.3)	205	56.3	81	50.9	91	40.8	
Moderate	198 (26.0)	90	24.7	43	27.0	62	27.8	
High	163 (21.4)	65	17.9	32	20.1	66	29.6	0.003
Attitude towards death								
Low acceptance	321 (42.2)	157	43.1	65	40.9	95	42.6	
Moderate acceptance	237 (31.1)	120	33.0	46	28.9	69	30.9	
High acceptance	177 (23.3)	78	21.4	45	28.3	51	22.9	0.56
Bioethics courses								
None	235 (31.4)	114	31.3	45	28.3	70	31.4	
≥ 1	514 (68.6)	246	67.6	110	69.2	150	67.3	0.81
Religion								
Believer	519 (68.2)	271	74.5	103	64.8	137	61.4	
Nonbeliever	237 (31.1)	93	25.6	55	34.6	86	38.6	0.003
Knowledge about IDD								
Low	317 (41.7)	147	40.4	67	42.1	100	44.8	
Intermediate	288 (37.8)	140	38.5	61	38.4	83	37.2	
Positive attitude and high knowledge	118 (15.5)	62	17.0	22	13.8	32	14.4	0.79
Knowledge about ASD								
Low	191 (25.1)	86	23.6	40	25.2	62	27.8	
Intermediate	303 (39.8)	161	44.2	65	40.9	75	33.6	
Positive attitude and high knowledge	224 (29.4)	98	26.9	45	28.3	77	34.5	0.11
Knowledge about ADHD								
Low	296 (38.9)	162	44.5	54	34.0	77	34.5	
Intermediate	247 (32.5)	100	27.5	56	35.2	87	39.0	
Positive attitude and high knowledge	173 (22.7)	87	23.9	40	25.2	44	19.7	0.01

^a^Not all percentages add up to 100%, due to missing values

^b^Chi^2^ test

As for predictors of low paternalism/autonomy, women were more likely to explicitly share diagnoses and therapeutic strategies with patients and their families (OR 1.57 [95% CI 1.11–2.22]) (Table 2). Similarly, another determinant of sharing-based autonomy (low paternalism) was having a specialty background (OR 1.67 [95% CI 1.16–2.40]). A pattern of open communication was strongly associated with low paternalism/autonomy (OR 12.13 [95% CI 7.71–19.05]). Among physicians with intermediate knowledge of ASD, the odds of low paternalism/autonomy were 0.60 (95% CI 0.40–0.91) compared with physicians having low knowledge about this disorder. Table 3 shows the association of communication attributes and specialty with low paternalism/autonomy in Mexico by age strata.

**Table 2:** Factors associated with low paternalism/autonomy of health professionals in Mexico, 2018

Variables	*n* (%)	OR^a^	Multiple^b^	
			95% CI	
Age				
43–76 years	229 (30.1)	1.0		
30–42 years	234 (30.8)	0.79	0.51	1.24
19–29 years	280 (36.8)	0.72	0.46	1.13
Gender				
Male	310 (40.7)	1.0		
Female	440 (57.8)	1.57	1.11	2.22
Specialty				
No	328 (43.1)	1.0		
Yes	425 (55.9)	1.67	1.16	2.40
Family member with IDD or ASD				
Yes	132 (17.4)	1.0		
No	630 (81.1)	0.98	0.64	1.50
Value assigned to truth (Truthful communication from the professional to the patient)				
Low	383 (50.3)	1.0		
Moderate	198 (26.0)	0.86	0.58	1.29
High	163 (21.4)	0.98	0.63	1.53
Communication pattern				
Withholding	315 (41.4)	1.0		
Partial communication	191 (25.1)	4.52	2.98	6.87
Open communication	230 (30.2)	12.13	7.71	19.05
Bioethics courses				
None	235 (31.4)	1.0		
≥ 1	514 (68.6)	0.76	0.53	1.11
Knowledge about IDD				
Low	317 (41.7)	1.0		
Intermediate	288 (37.8)	0.75	0.52	1.08
Positive attitude and high knowledge	118 (15.5)	0.62	0.39	1.01
Knowledge about ASD				
Low	191 (25.1)	1.0		
Intermediate	303 (39.8)	0.60	0.40	0.91
Positive attitude and high knowledge	224 (29.4)	1.04	0.67	1.63
Knowledge about ADHD				
Low	296 (38.9)	1.0		
Intermediate	247 (32.5)	1.27	0.85	1.89
Positive attitude and high knowledge	173 (22.7)	0.93	0.61	1.43

^a^Odds ratio, ordinal logistic regression

^b^Odds ratio, adjusted by all variables included in table, participant institution (medical facility/university) and religion

**Table 3:** Association of communication attributes and specialty with low paternalism/autonomy in health professionals: age modifying effect in Mexico, 2018

Variables	19–29 years			30–42 years			43–76 years			*p* value
	OR^a^	95% CI		OR^a^	95% CI		OR^a^	95% CI		
Communication pattern										
Withholding	1.0			1.0			1.0			
Partial communication	2.73	1.40	5.32	10.23	4.46	23.51	5.57	2.49	12.49	
Open communication	11.10	5.31	23.10	26.26	10.43	66.10	11.48	4.68	28.15	
Interaction term										< 0.001
Specialty										
No	1.0			1.0			1.0			
Yes	0.94	0.54	1.63	2.87	1.24	6.64	2.03	0.98	4.21	
Interaction term										0.03

^a^Odds ratio adjusted by gender, family member with IDD or ASD, value assigned to truth, bioethics courses, religion, participant institution (medical facility/university), and knowledge about IDD, ASD, and ADHD

## Discussion

In the present study, out of 761 mental health professionals, 29.3% displayed a low paternalistic/autonomist attitude, while the rate of high paternalism/overprotection was 47.8%. Our results are consistent with the paternalistic model proposed by Charles et al. [9, 33], in which patients rely on physicians to make treatment decisions rather than using a more collaborative process, as well as with other research studies [12, 34–37]. In contrast, in a study of 1,050 US physicians, 75% preferred a model based on shared decision-making (understood as “an approach in which physicians and patients share the best available evidence when faced with the task of making decisions, and where patients receive support, while considering their options, to achieve informed preferences” [38]), and only 14% preferred a paternalistic communication model [20].

In recent decades, the doctor–patient relationship has moved from a paternalism-based model to one that advocates respect for patient autonomy; however, this transition has not been uniform throughout the world. Countries have different cultural, historical and political determinants that can influence the speed and manner in which this transition occurs [39]. In addition, some professionals have doubts about the implementation of an autonomist model based on sharing decisions, saying that their patients do not want to participate in decision-making, do not have the capacity to participate or could even make poor decisions, and considering that consultations take time that they do not have [38]. According to our results, a study among Mexican physicians involved in long-term care indicated that they made treatment decisions instead of leaving decisions to patients [27]. Therefore, it is clear that the first step toward a more participatory, more autonomous model is to ensure that physicians have developed the necessary skills for implementing this model and to evaluate the impact on patient’s health.

Considering the dynamics of the doctor–patient relationship, it is important to take into account that some patients modify their communication style with their doctors depending on the severity of their disease and prefer to leave decisions to health professionals [40]; additionally, it has been shown that patients’ communication styles are also related to factors such as age, gender or origin [41–44]. In Mexico, consistent with findings for other countries [42, 43, 45], patients have reported that they prefer to play a passive role during consultations [46]. Conversely, a study carried out in the United Kingdom indicated that most patients would rather get as much information as possible about their health status to make informed decisions [47]. Similarly, a study in Japan mentions that a low proportion of patients (17%) prefer to leave decisions to their relatives or their doctors [48]. In multiple scenarios, patients report that they need to feel that their doctors care about them and listen to them [49–51] and that they are interested in participating in decisions because it makes them feel valued as humans [50]; even in clinical settings involving palliative care, patients prefer a communication style in which respect for autonomy prevails [52]. In this sense, in our study of mental healthcare professionals, we observed that open communication was associated with low paternalism/autonomy. Several studies and measures have been developed to assess physician communication patterns. The literature has documented that adequate communication allows direct discussion of health problems; e.g., to communicate diagnosis and treatment plans, and thus helps to establish positive and healthy relationships between doctors and patients [53–56].

A determining factor for the communication process is the value that the doctor places on truth, which is a human quality that develops throughout life [57, 58]. In our study, we did not find that the value assigned to truth was statistically associated with low paternalism/autonomy. In this context, respect for autonomy requires that patients be adequately and appropriately informed about their diagnosis, treatment options and prognosis. While there are extreme precedents that suggest that the value of life and survival nullifies the value of truth [3], this clearly is not a general principle in medical practice. In an conservative environment with deeply rooted cultural values, paternalism is accentuated, and the possibilities of autonomy and patient empowerment [59] are not considerations. Despite the discomfort and uncertainty that communicating a diagnosis or a poor prognosis can evoke, physicians should have to tell the truth.

In this study, women had a more autonomist attitude/lower paternalism than men. Gender differences in practice and communication style have been well documented in the literature [22, 60, 61]. In line with the practice of a more autonomist style, a study that assessed psychiatrists’ sharing-based decision behaviours reported higher scores for women [62]. Likewise, in more recent studies, female physicians [21, 63] and medical students [63, 64] had significantly more patient-centred attitudes (understood as sharing power, control, and information; respect for patients’ feelings, expectations, and preferences taking these factors into account in medical decision-making [65]), suggesting that gender-stereotyped communication is established through medical students’ attitudes and it seems to persist among practicing physicians [63]. These differences are even more important in that they lead to corresponding differences in patients’ behaviour towards physicians. Results from a meta-analysis on the effects of physician gender on patient communication suggested that physicians’ behaviour is largely mirrored back in the behaviour shown to them by patients [66]. Previous research on doctor–patient communication has revealed that female physicians conduct longer consultations [61] and engage in more partnership behaviours and discussions about psychosocial aspects, and they communicate a higher degree of empathy [21, 61, 67]. Such patient-centred care may result in better health outcomes [68]; however, due the increasing burden of mental disorders and its recent inclusion in the United Nations’ Sustainable Development Goals [69], reducing these gender differences is essential to covering the healthcare demand for these diseases.

We also found that having a specialty was associated with low paternalism/autonomy; however, other studies have shown that even when doctors specialize, the paternalistic attitude persists with differences by specialty. In a study of 104 German physicians, Falkum and Førde found that psychiatrists had significantly lower paternalism scores than physicians with somatic specialties and specialists in social medicine, possibly because an understanding of both the cognitive and emotional aspects of the doctor–patient relationship is considered crucial to the practice of psychiatry [19]. Results from a cross-sectional survey of US physicians indicated that professionals with a medical specialty were more likely to prefer paternalism than professionals in primary care (including family practice, general practice, internal medicine and paediatrics), while surgeons were the least likely to prefer it [20]. A more recent study conducted in Japan, Taiwan, and Korea to assess physicians’ attitudes toward patient autonomy showed that, compared with physicians in internal medicine, those specializing in surgery were significantly more likely to agree with the statement that the patient should be told the truth, even if the family disagrees [23]. A qualitative study conducted to analyse the perspectives of US paediatricians regarding shared decision-making in ADHD showed that instead of familiarizing families with all the options first, the paediatricians provided information to persuade families to accept the treatment of their choice [70]. These different scenarios can be explained in part by the quality of communication between the physician and the patient/parent, the technical language used, and possibly the cultural context of both parties [71] as well as the development of medical bioethics and doctor–patient communication skills [72].

Professionals’ limited knowledge about mental health and stigmatizing attitudes toward mental illness can delay the diagnosis of autism [73] and other disorders, such as IDD (a so-called intellectual disability), which can result in inequitable access to healthcare services [74] perhaps due to a poor communication process. In our study, the percentage of mental health professionals with a positive attitude toward and high knowledge about ASD and ADHD was 29.4% and 22.7% respectively, while that for IDD was lower (15.5%); these results could be explained by the fact that IDD is not clearly perceived as a pathological condition and, to an even greater degree, it is not considered a public health problem in Mexico. In the global north, insufficient training in IDD management has been reported [75, 76]. A study conducted on a sample of nurses working at an emergency department showed that although the majority reported interacting with a patient with suspected intellectual disability in the past year, only 28% of those surveyed considered themselves to have the knowledge to determine that a patient may have an intellectual disability, and only half reported feeling capable of adopting adequate communication [77]. There are few studies evaluating the level of ASD knowledge among physicians screening children in the general population [78]. In a study implemented to evaluate the knowledge of ASD among 93 Dutch physicians who screened children for psychiatric symptoms, the results showed a general ASD knowledge score of 7.1 (SD 1.2) on a 1–10 scale but a specific ASD knowledge score of only 5.7 (SD 1.7) [73]. For ADHD, a cross-sectional study of 340 primary health physicians in Saudi Arabia that aimed to collect data on personal characteristics, knowledge, attitude, and diagnosis and management practices in connection to this disorder revealed that approximately one-third of physicians had a poor level of knowledge [79]. Similar to our study, some studies have used a questionnaire based on clinical vignettes with multiple-choice response, while other studies have used a range of different assessment approaches (e.g., checklists, short answers, interviews, true/false or Likert-scale response options) [78]; however, there is little consensus as to the most precise method. As different instruments are used to evaluate knowledge level, it could be difficult to assess whether and how the level of mental illness knowledge among Mexican psychiatrists and neurologists compares with that of health professionals in other countries. An adequate level of knowledge that allows early detection and initiation of treatment is an important factor to optimize development and improve the lifelong outcomes of people with autism [80, 81] and other neurodevelopmental disorders.

Finally, in our study, we found that the association between communication patterns and specialty and paternalism was modified by health professionals’ age, and this effect was greatest among professionals aged 30 to 42 years. A study of Israeli doctors reported that younger doctors communicated better than older ones [82]. A multi-country study found that doctors under 40 years of age have a more proactive attitude toward discussing an unfavourable prognosis [83]. According to Honeycutt et al., paediatricians and younger doctors are more likely to prefer a participatory style with parents of ADHD children [84]; this may be due in part to medical school programs in US making efforts to include a humanistic approach and seminars on doctor–patient communication [84].

## Limitations

Due to the nature of the design, we reported associations and did not consider causal relationships. Furthermore, this study used a convenience sample, which can compromise the generalisation of the results. Although results do not represent all health professionals, they may provide the first situational description of the determinants of paternalism in communication patterns between health professionals and parents of patients with neurodevelopmental disorders or other diseases.

## Conclusions and recommendations

A considerable pattern of paternalism/overprotection prevailed among mental health professionals in Mexico. Gender, specialty, and a pattern of open communication were closely associated with low paternalism/autonomy. Discussions on the communication patterns that doctors use with their patients in Mexico aim to establish, without judgment, the importance of creating a more effective relationship between physicians and their patients by (a) reinforcing the practice of patient-centred medicine, (b) ensuring that professionals develop the highest level of medical competencies, including ethical values and doctor–patient communication skills, while considering physicians’ personal and professional characteristics, and (c) promoting autonomy in the doctor–patient relationship as we strive for a society in which self-determination is not considered a privilege but a human right—that is, a society characterized by social justice. It is also necessary for health professionals to develop strategies that facilitate shared decision-making. Such strategies include providing clear and simple explanations, verifying understanding, listening to patients’ concerns and needs, reaching a consensus with patients regarding the treatment plan and establishing a follow-up plan that is convenient for both parties [85]. More research is needed to provide evidence as to which mode of care is more beneficial and fitting in each context, particularly in the relationship with parents of patients with certain neurodevelopmental disorders.

## Data Availability

The datasets used and/or analysed during the current study are available from the corresponding author upon reasonable request.

## Abbreviations

OR(s): Odds ratio(s)
CI(s): Confidence interval(s)
IDD: Intellectual development disorder
ASD: Autism spectrum disorder
ADHD: Attention deficit hyperactivity disorder
